# Gradients of O-information highlight synergy and redundancy in physiological applications

**DOI:** 10.3389/fnetp.2023.1335808

**Published:** 2024-01-09

**Authors:** Tomas Scagliarini, Laura Sparacino, Luca Faes, Daniele Marinazzo, Sebastiano Stramaglia

**Affiliations:** ^1^ Dipartimento di Fisica e Astronomia G. Galilei, Università degli Studi di Padova, Padova, Italy; ^2^ Dipartimento di Ingegneria, Università di Palermo, Palermo, Italy; ^3^ Department of Data Analysis, Ghent University, Ghent, Belgium; ^4^ Dipartimento Interateneo di Fisica, Università degli Studi di Bari Aldo Moro, Bari, Italy; ^5^ Center of Innovative Technologies for Signal Detection and Processing (TIRES), Università degli Studi di Bari Aldo Moro, Bari, Italy

**Keywords:** information theory, high-order interactions, network science, functional brain connectivity, cardiovascular interactions, cardiorespiratory interactions, cerebrovascular interactions

## Abstract

The study of high order dependencies in complex systems has recently led to the introduction of statistical synergy, a novel quantity corresponding to a form of emergence in which patterns at large scales are not traceable from lower scales. As a consequence, several works in the last years dealt with the synergy and its counterpart, the redundancy. In particular, the O-information is a signed metric that measures the balance between redundant and synergistic statistical dependencies. In spite of its growing use, this metric does not provide insight about the role played by low-order scales in the formation of high order effects. To fill this gap, the framework for the computation of the O-information has been recently expanded introducing the so-called gradients of this metric, which measure the irreducible contribution of a variable (or a group of variables) to the high order informational circuits of a system. Here, we review the theory behind the O-information and its gradients and present the potential of these concepts in the field of network physiology, showing two new applications relevant to brain functional connectivity probed via functional resonance imaging and physiological interactions among the variability of heart rate, arterial pressure, respiration and cerebral blood flow.

## 1 Introduction

Two of the fields of complex systems science which are experiencing an increasing interest in the last years are (i) the analysis of high order interactions and (ii) the decomposition of multivariate information in redundant and synergistic contributions.

High order interactions represent the structural organization of couplings, in a complex system, where interactions may involve groups of three or more units. Indeed, high order structures, such as hypergraphs and simplicial complexes, are thought to be better tools than dyadic networks to map the real organization of many social, biological and man-made systems (Battiston et al., 2020; Battiston et al., 2021). On the other hand, the decomposition of multivariate information in redundant and synergistic contributions is related to the joint probability distribution of the system, evaluated exploiting samples from its dynamics, and speaks to the properties of the marginal probabilities of groups of variables. Since the pairwise description has been found to be insufficient for explaining the orchestrated information flow among multiple components of complex systems, the quantification of high order statistical dependencies attracted the attention of a large community (Crutchfield, 1994; Bettencourt et al., 2008; Stramaglia et al., 2012). In the language of information theory, redundancy occurs when multiple copies of the same information can be found in different parts of a group of variables, while synergy refers those information which is not stored in any specific element, but rather in the joint state of that group of variables.

These two lines of research are actually complementary and realize, at the level of high order phenomena, the dualism structure-function. The former focuses on the structural organization of a system in terms of structural hyperlinks, the latter on emergent properties related to what the system *does*, and characterises its high order behavior identifying an equivalent to functional hyperlinks from data sampled at nodes (Rosas et al., 2022). The present work deals with the second perspective, studying high order dependencies in data obtained from complex systems.

The emergence of new tools for the quantification of high order interactions is opening new possibilities in the field of network physiology, which aims to address the fundamental question of how physiological networks collectively behave to maintain human body in healthy conditions (Bashan et al., 2012; Lin et al., 2020; Ivanov 2021). Historically, the study of physiological time series has seen a shift from the univariate analysis of individual time series, where measures such as the approximate entropy (Pincus, 1991), the sample entropy (Richman and Moorman, 2000) and the corrected conditional entropy (Porta et al., 1998) have been introduced to characterize the predictable dynamics of a physiological system, to the bivariate analysis of two time series, where symmetric or causal measures based on cross-entropies (Porta et al., 1999; Faes et al., 2011), mutual information (Valderas, 2019) and its rate (Barà et al., 2023), directed information (Massey, 1990) or transfer entropy (Schreiber, 2000; Faes et al., 2014) have been used extensively to quantify the information shared and transferred between pairs of physiological systems. Multivariate analyses involving more than two physiological time series have been then introduced to quantify how the information transferred between processes is affected by the rest of the network. (Montalto et al., 2014; Wang et al., 2022). However, the multivariate approach has been implemented largely to analyze pairwise interactions between two systems while accounting for the presence of other systems, rather than to investigate how several systems interact collectively to shape the network dynamics. For this reason, high order interactions have not been studied explicitly in physiological networks until very recently (Stramaglia et al., 2021), and thus remain a tool whose potential in the field of network physiology is poorly addressed.

The major approach for estimating synergy and redundancy from data is partial information decomposition (PID) (Williams and Beer, 2010; Wibral et al., 2017; Lizier et al., 2018): applications in neuroscience have shown that the description of the brain dynamics in terms of synergy and redundancy (Luppi et al., 2022; Varley et al., 2023) is particularly suited to the interplay between brain segregation and integration (Bassett and Sporns, 2017). Moreover, it has also been used in different physiological contexts, such as to dissects control mechanisms of heart rate variability at rest and during physiological stress (Krohova et al., 2019); however, the use of PID in many applications is greatly limited by the super-exponential growth of decomposition terms for large systems. To cope with the computational burden of PID, but giving up the possibility of independently evaluating synergy and redundancy, the O-information has been introduced in (Rosas et al., 2019) as a metric measuring the balance between synergy and redundancy and thus being capable of characterising synergy-dominated systems: its computational weight scales gracefully with the size of systems. In (Scagliarini et al., 2022) the local O-information has been proposed to study inter-dependencies on individual patterns. A new framework for the time- and frequency domain assessment of high order interactions in networks of random processes has been developed in (Faes et al., 2022).

To complement the global assessment provided by the O-information, it has been recently proposed to exploit the gradients of the O-information as low-order descriptors that can characterize how high order effects are localized across a system of interest (Scagliarini et al., 2023). Instead of focusing on the O-information of groups of variables, the attention here is focused on the variation of the O-information when variables are added to the rest of the system to form these groups. This provides a more nuanced description of synergistic or redundant informational circuits, in which the role of each variable can be disambiguated. In this work, we first review the approach for the computation of the O-information and its gradients, and then present two new applications related to network physiology: (i) fMRI data from healthy subjects in resting conditions and (ii) cardiovascular, cerebrovascular and respiratory oscillations in healthy subjects in the supine resting state and after head-up tilt.

## 2 Gradients of O-information

In this section, we recall the definition and the properties of the gradients of O-information. First of all, it is useful to introduce O-information, which measures the balance between redundancy and synergy, representing the two basic types of high order statistical dependencies. The two building blocks of O-information are the total correlation TC (Watanabe, 1960) and the dual total correlation DTC (Sun, 1975), defined as follows for a system described by *n* stochastic variables ***X*** ^*n*^ = {*X*_1_, *…*, *X*_*n*_}:

$$\begin{array}{ll} TC\left(X^{n}\right) & ≔\overset{n}{\underset{i=0}{\sum }}H\left(X_{i}\right)-H\left(X^{n}\right),\\ DTC\left(X^{n}\right) & ≔H\left(X^{n}\right)-\overset{n}{\underset{i=0}{\sum }}H\left(X_{i}|X_{-i}^{n}\right), \end{array}$$

where $X_{-i}^{n}$ denotes the set of all the variables in ***X*** ^*n*^ but *X*_*i*_, *H* is the Shannon entropy and *H*(*X*_*i*_|*X*_*j*_) = *H*(*X*_*i*_, *X*_*j*_) − *H*(*X*_*j*_) is the conditional Shannon entropy. TC quantifies the collective constraints, whilst DTC quantifies the shared randomness. The O-information is defined as the difference TC-DTC and assumes positive values when the interdependencies among variables can be more efficiently explained as shared randomness, and negative values when collective constraints can be more convenient (Rosas et al., 2019).

Consequently, the O-information of the system can be written as (Rosas et al., 2019)

$$\Omega \left(X^{n}\right)=\left(n-2\right)H\left(X^{n}\right)+\overset{n}{\underset{i=1}{\sum }}\left[H\left(X_{i}\right)-H\left(X_{-i}^{n}\right)\right].$$ (1)

If *Ω* > 0, the system is redundancy-dominated. On the other hand, when *Ω* < 0 the dependencies are better explained as patterns that can be observed in the joint state of multiple variables but not in subsets of these; in other words, the system is synergy-dominated. It is clear that the main drawback of the O-information is the fact it does not put in evidence multiplets of variables which are both redundant and synergistic with equal strength, whilst approaches like PID evaluate both quantities and may, in principle, deal with these cases. It is also worth mentioning that the O-information is connected to the Synergy-Redundancy Index (SRI) developed in (Gat and Tishby, 1998; Brenner et al., 2000; Reich et al., 2001; Puchalla et al., 2005); although the SRI does not provide separate quantifications of synergy and redundancy like PID does, it can consider the respective contributions of signal correlations and noise correlations to synergy and redundancy (Panzeri et al., 1999; Nirenberg and Latham, 2003; Latham and Nirenberg, 2005; Panzeri et al., 2022). Therefore, SRI should be regarded as complementary to PID/O-information approaches.

In order to measure how much a given variable *X*_*i*_ plays a role in the informational circuits contained in ***X*** ^*n*^, its “gradient of O-information” is calculated as follows (Scagliarini et al., 2023):

$$\begin{array}{ll} \partial_{i}\Omega \left(X^{n}\right) & =\Omega \left(X^{n}\right)-\Omega \left(X_{-i}^{n}\right)\\ & =\left(2-n\right)I\left(X_{i};X_{-i}^{n}\right)+\overset{n}{\underset{k=1,k\neq i}{\sum }}I\left(X_{k};X_{-ik}^{n}\right), \end{array}$$ (2)

where *I* is the mutual information and $X_{-ik}^{n}$ denotes all the variables in ***X*** ^*n*^ except *X*_*i*_ and *X*_*k*_. The quantity *∂*_*i*_Ω(***X*** ^*n*^) captures how much the O-information changes when *X*_*i*_ is added to the rest of the system, hence it gives an account of how this variable contributes to the high order properties of the system. Therefore, *∂*_*i*_Ω(***X*** ^*n*^) > 0 means that *X*_*i*_ introduces mainly redundant information, while *∂*_*i*_Ω(***X*** ^*n*^) < 0 indicates that it fosters synergistic interdependencies.

It has been shown in (Scagliarini et al., 2023) that the following bounds hold and are tight:

$$-\left(n-2\right)\log|X|\leq \partial_{i}\Omega \left(X^{n}\right)\leq \log|X|,$$ (3)

where |X| is the cardinality of the largest alphabet in ***X*** ^*n*^. The lower bound is achieved in correspondence of the *n*-XOR gate, that is *X*_1_…*X*_*n*−1_ as Bernoulli random variables with *p* = 1/2 and $X_{n}=\left(\sum_{j=1}^{n-1}X_{j}\right)\;\text{mod}\;2$ ; the upper bound is achieved by the *n*-COPY gate, specifically by taking *X*_1_ as a Bernoulli variable with *p* = 1/2 and *X*_1_ = *X*_2_ = ⋯ = *X*_*n*_. The asymmetry between these two bounds has the following consequence: while redundancy can be only built step by step, synergy can be established more rapidly. Indeed, adding a variable to a system of size *n* − 1 might provide a maximal redundant contribution of log|X| , whilst the maximal synergy that it might lend is (n−2)log|X| — which can be substantial if *n* is large.

Following a similar rationale to the one that leads to Eq. 2, one can further introduce a second-order descriptor of high order interdependencies by considering gradients of gradients. In particular, the second-order gradient of a pair of variables *X*_*i*_ and *X*_*j*_ can be defined as

$$\partial_{ij}\Omega \left(X^{n}\right)=\partial_{i}\Omega \left(X^{n}\right)-\partial_{i}\Omega \left(X_{-j}^{n}\right).$$ (4)

This second-order gradient captures how much the presence of the variable *X*_*j*_ alters the variation of O-information of the system due to the inclusion of *X*_*i*_. It is direct to verify the symmetry *∂*_*i*_ *∂*_*j*_Ω(***X*** ^*n*^) = *∂*_*j*_ *∂*_*i*_Ω(***X*** ^*n*^); therefore, we simply denote this quantity as $\partial_{ij}^{2}\Omega \left(X^{n}\right)$ . An interesting property of $\partial_{ij}^{2}\Omega \left(X^{n}\right)$ is that it can be re-written as a ‘whole-minus-sum’ property:

$$\begin{array}{llll} \partial_{ij}^{2}\Omega \left(X^{n}\right) & =\left[\Omega \left(X^{n}\right)-\Omega \left(X_{-ij}^{n}\right)\right] & - & \left[\Omega \left(X_{-i}^{n}\right)-\Omega \left(X_{-ij}^{n}\right)\right]-\left[\Omega \left(X_{-j}^{n}\right)-\Omega \left(X_{-ij}^{n}\right)\right]. \end{array}$$ (5)

In other words, $\partial_{ij}^{2}\Omega \left(X^{n}\right)$ measures to what degree the variation to the O-information due to the inclusions of both *X*_*i*_ and *X*_*j*_ is more than the sum of the variations one obtains when including them separately. It is interesting to evaluate $\partial_{ij}^{2}\Omega \left(X^{n}\right)$ on the *n*-COPY gate and on the *n*-XOR gate: it is easy to obtain zero and (2 − *n*) respectively. This means that for the *n*-COPY gate pairs of variables do not provide further redundancy w.r.t. those provided by single variables; on the other hand, for the *n*-XOR gate, pairs of variables give an irreducible contribution to the synergy. This is a sign of the sensitivity of gradients to evaluate synergistic informational circuits, which occurs due to the partition of $X_{-ij}^{n}$ into parts which is adopted to calculate them.

Successive gradients can be similarly introduced, resulting in a simple chain rule. If *γ* is a subset of {1, …, *n*} of cardinality |*γ*|, then:

$$\partial_{\gamma }^{\left|\gamma \right|}\Omega \left(X^{n}\right)=\underset{\alpha \subseteq \gamma }{\sum }{\left(-1\right)}^{\left|\alpha \right|}\Omega \left(X_{-\alpha }^{n}\right),$$ (6)

the sum being over all the subsets *α* of *γ*. For example, for triplets of variables the gradient of the O-information reads:

$$\begin{array}{lll} \partial_{\mathrm{ijk}}^{3}\Omega \left(X^{n}\right) & =\Omega \left(X^{n}\right)-\Omega \left(X_{-i}^{n}\right)-\Omega \left(X_{-j}^{n}\right)-\Omega \left(X_{-k}^{n}\right)+ & \Omega \left(X_{-ij}^{n}\right)+\Omega \left(X_{-ik}^{n}\right)+\Omega \left(X_{-jk}^{n}\right)-\Omega \left(X_{-ijk}^{n}\right), \end{array}$$ (7)

and measures the irreducible contribution to the O-information by the triplet {*i*, *j*, *k*} which cannot be ascribed to the inclusion of pairs nor single variables of the triplet.

## 3 Application to physiological networks

This section reports the application of the measures defined in Section 2 to physiological networks where high order interactions are expected to play a role in the generation of the network dynamics, i.e., brain networks probed by functional magnetic resonance imaging (fMRI) and networks of cardiovascular and cerebrovascular interactions probed by the beat-to-beat variability series of cardiac, vascular, respiratory and cerebral blood flow parameters. Gradients of O-information were calculated using the Gaussian Copula approach described in (Ince et al., 2017) to estimate entropy terms.

### 3.1 Brain networks: fMRI data

We first consider the data from the Human Connectome Project (Van Essen et al., 2012) corresponding to 1,083 healthy subjects whose organization of networks in the human cerebrum was explored using resting-state functional connectivity MRI (Yeo et al., 2011). The fMRI data acquisitions have been performed on a Siemens 3T Skyra scanner at Washington University (WashU). In order to construct a best-estimate parcellation of the human cerebral cortex to serve as a reference for future studies, a clustering algorithm was used to parcellate the cerebral cortex into networks of functionally coupled regions. Parcellations were examined for a coarse solution that organized the cortex into seven networks as well as a finer solution that identified 17 networks. The estimated networks were found to be consistent across the discovery and replication data samples and were confirmed by region-based functional connectivity MRI (fcMRI) analyses. Here we consider the parcellation in 7 clusters, each corresponding to the following connectivity networks: Default, Control, Limbic, Visual, Somatosensor, Ventral Attention, and Dorsal Attention. For each subject, we analyze the corresponding seven fMRI time series. The significance of the detected high order interactions is assessed using the statistics of subjects: gradients are considered significantly redundant (synergistic) when the 5-th (95-th) percentile of the distribution is higher (lower) than zero.

**FIGURE 1:**
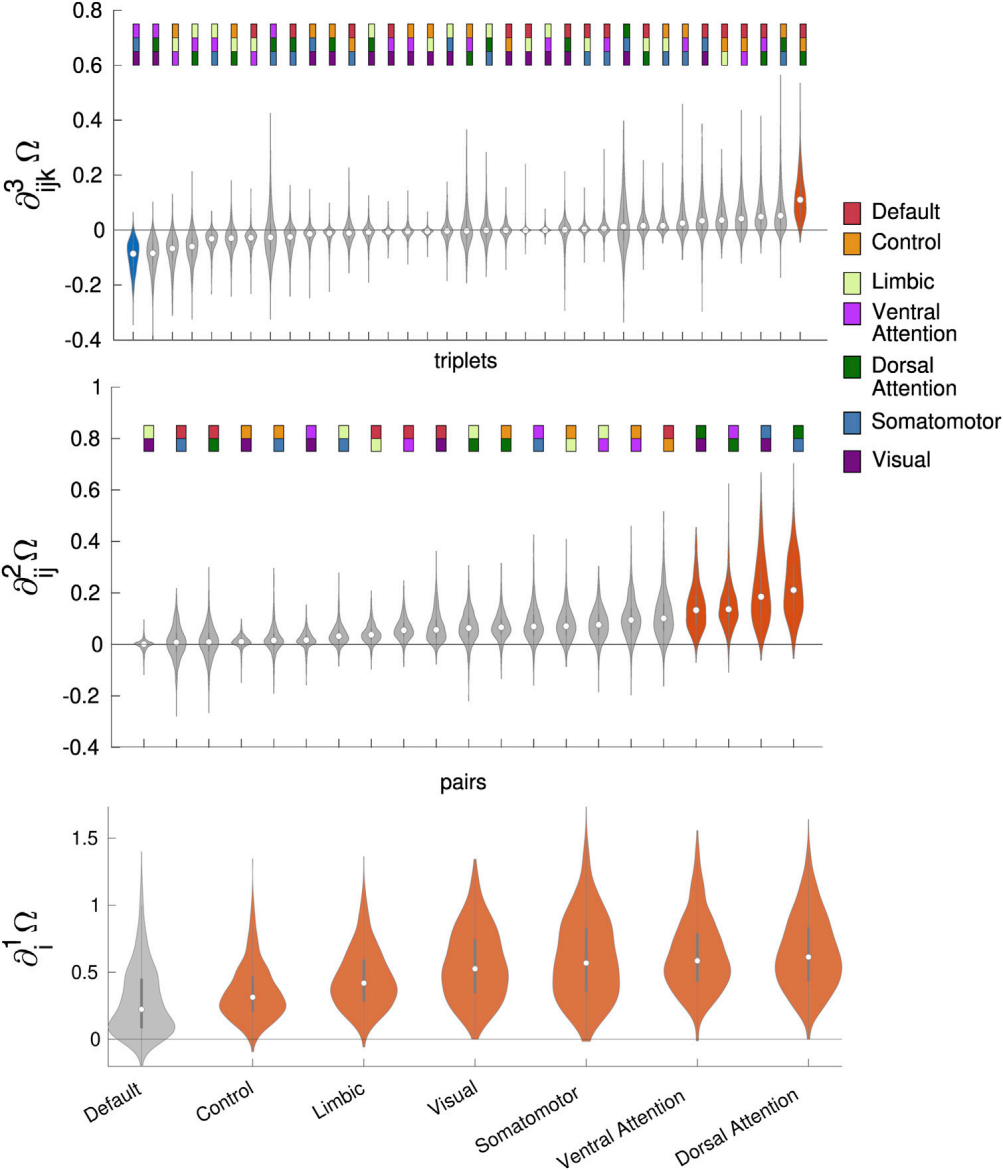
First order gradients of the O-information (bottom) for the seven fMRI time series of resting state brain networks. Six out of seven are significantly redundant. Signals from the Default Mode Network (DMN) are not significantly redundant, hence suggesting that the DMN it is the region for whom the balance synergy-redundancy is less leaning towards redundancy. The second-order (middle) and third-order (top) gradients of the O-information for the 21 pairs and the 35 triplets of fMRI time series of resting state brain networks. Colored rectangles represent the composition of the pairs and the triplets in terms of the resting state networks shown in legend. Redundant and synergistic violins are depicted in red and blue, respectively. Four pairs are significantly redundant. One triplet is significantly synergistic and one is significantly redundant.

In bottom panel of Figure 1 we depict the first-order gradient computed for the seven intrinsic connectivity networks. Except for the Default, all the regions are significantly redundant. Going to the second order gradients (middle panel of Figure 1), four pairs of regions are significantly redundant: somatomotor—dorsal attention, somatomotor—visual, dorsal attention—ventral attention, dorsal attention—visual. Concerning third order gradients, we find a significantly redundant triplet, default—control—dorsal attention, and a significantly synergistic triplet: ventral attention-somatomotor—visual (see top panel of Figure 1). These results evidence peculiar intrinsic connectivity networks contributing to redundancy and synergy in the large-scale organization of the overall fMRI network, and confirm that gradients of increasing order tend to highlight less redundant/more synergistic interactions.

### 3.2 Multi-organ networks: cardiovascular, respiratory and cerebral blood flow variability

In the second application, we analyze a database of physiological time series collected to study the effect of postural stress on cardiovascular, cerebrovascular and respiratory variability (Faes et al., 2013; Bari et al., 2016). The original dataset is comprised of 13 healthy subjects (age: 27 ± 8 years; 5 males), enrolled at the Neurology Division of Sacro Cuore Hospital, Negrar, Italy. Electrocardiogram (ECG, lead II) was acquired together with arterial pressure (AP) measured at the level of middle finger through a photopletysmographic device (Finapres Medical Systems, Ohmenda, Netherlands). Cerebral blood flow velocity (CBFV) and respiration were measured at the level of the middle cerebral artery by means of a transcranial Doppler ultrasonographic device (Multi-Dop T2, Dwl, San Juan Capistrano, CA) and through a thoracic impedance belt, respectively. Signals were synchronously acquired at a sampling rate of 1 kHz. From the raw signals, the physiological beat-to-beat variability series of heart period (H), systolic AP (S), mean AP (M), mean CBFV (F) and respiration (R) were measured as detailed in (Faes et al., 2013; Bari et al., 2016) during two stationary time windows of length 250 beats in the following physiological conditions: (i) supine rest (REST) and (ii) head-up tilt test with table inclination of 60° (TILT). Prior to network analysis, each series was high-pass filtered to remove slow trends and normalized to zero mean and unit variance.

**FIGURE 2:**
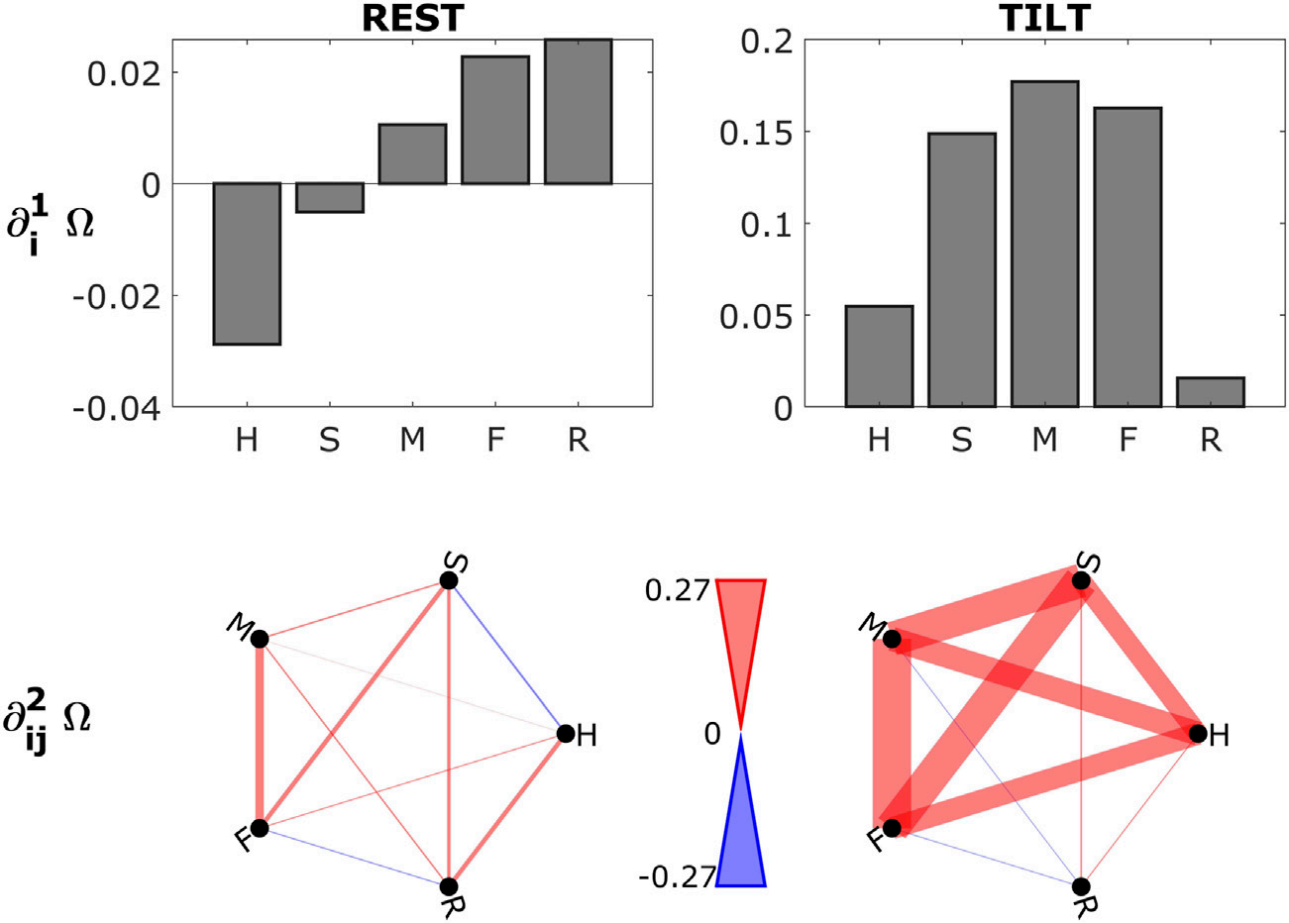
First (top row) and second (bottom row) order gradients for the physiological system composed of the five time series {*H*, *S*, *M*, *F*, *R*}, averaged over subjects and computed in the two experimental conditions: supine resting state (REST, left plots) and head-up tilt (TILT, right plots). Colours indicate redundant (red) and synergistic (blue) characters of interaction. Width of the links indicates the strength of the gradients. Statistical significance was assessed via bootstrap data analysis for each subject. As regards the first order gradients in the resting state, one out of the five showed significant synergy in 50% of subjects (H), while in TILT significant redundancy was found for three of the five (S, M, F). Going to the second order gradients, only in the TILT condition redundancy was significant in more than 50% of subjects for the pairs H-M, H-F, S-M, S-F, M-F. This suggests an important role of the sympathetic activation led by head-up tilt in increasing redundancy in physiologic networks.

The first and second order gradients evaluated for the physiologic network constituted by the five time series {*H*, *S*, *M*, *F*, *R*} (average over subjects) are reported in Figure 2. Bootstrap data analysis was applied to assess the statistical significance of the computed measures for each subject: gradients are considered significantly redundant (synergistic) when the 5-th (95-th) percentile of the bootstrap distribution is higher (lower) than zero. Looking at the first order gradients (Figure 2, top row), we see that the heart plays a synergistic role in the resting state, whilst in the orthostatic position the system becomes dominated by redundancy with a disconnection of respiration. The analysis of the second order gradients provided similar results (Figure 2, bottom row), with the cardiovascular link between H and S showing a synergistic character during the supine rest, and with increasing redundant behavior of the whole network after head-up tilt.

## 4 Discussion

In this work, we have shown that recently proposed computational techniques show the ability to find multiplets of synergistic variables in physiological applications without requiring a huge amount of data.

In fMRI, data motion and physiological noise contribute substantially to the overall system variance (Liu et al., 2017; Colenbier et al., 2020). Redundancy is thus the first quantity to be naturally reduced at lower orders. In (Luppi et al., 2022) the synergistic and redundant districts of the resting brain have been explored, and it has been found that redundant interactions are especially prominent in the primary sensory, primary motor and insular cortices, corresponding to the brain’s somatomotor and salience subnetworks. In contrast, regions with higher relative importance for synergy predominate in high order association cortex, and are affiliated with the default mode (DMN) and fronto-parietal executive control (FPN) subnetworks. We note that in Varley et al. (2023) an analogous synergy-redundancy gradient as in Luppi et al. (2022) has been found using partial entropy decomposition.

It is worth mentioning that in (Luppi et al., 2022) dynamical synergy and redundancy (from the double redundancy lattice, (Mediano et al., 2021), have been explored for each pair of the 232 regions of the augmented Schaefer atlas. In agreement with (Luppi et al., 2022), we find that the default network has the minimum first order gradient, i.e., it is the less redundant; moreover a major redundant role is played by the somatosensor network. However our results refer to a different spatial scale and, come from a static analysis, are not expected to be fully reproducing with the results in (Luppi et al., 2022): notice that the emergence of a synergistic circuit made of visual, somatomotor and ventral attention has not been observed in previous studies. We remark that in a recent paper it has been observed that ventral attention and motor network connectivity are relevant to functional impairment in spatial neglect after right brain stroke (Barrett et al., 2019); moreover higher functional connectivity of ventral attention and visual network has been found to play a role to maintain cognitive performance in white matter hyperintensity (Zhu et al., 2023). These findings renders even more interesting our results, i.e., these three networks belonging to a synergistic informational circuit in the resting brain.

Our results also document a well known fact in physiology, i.e., that cardiovascular, cerebrovascular and respiratory interactions are highly redundant. On the other hand, we also show an interesting aspect of cardiovascular oscillations, i.e., that the heart rate plays a synergistic role in the resting state analyzed with our static analysis; synergy could result from the fact that heart rate variability is the target for several neuro-autonomic mechanisms including the cardiac baroreflex and the respiratory sinus arrhythmia (Cohen and Taylor, 2002). We also find that redundancy is strongly enhanced by the entrainment of cardiovascular and cerebrovascular oscillations and by sympathetic activation; in particular, in the upright position all the series are highly redundant, except for the respiration signal, which is out of the redundant circuits in tilt conditions. These results agree with the tilt-induced shift of the sympatho-vagal balance towards increased sympathetic activity and decreased parasympathetic activity (Montano et al., 1994), also previously documented via information-theoretic analyses (Faes et al., 2011). Overall, the redundancy showed also a tendency to increase with tilt, documenting an effect of sympathetic activation on the redundant interactions among cardiovascular and cerebrovascular oscillations (Faes et al., 2022). Bootstrap data analysis (Politis, 2003) confirmed these findings, suggesting that interactions involving respiratory, arterial pressure and blood flow variabilities are more shifted to synergistic rather than redundant modes of interplay, as well as that significance increases moving from the supine to the upright position, confirming that redundancy is significantly strongly enhanced during the orthostatic stress.

Summarizing, gradients of O-information constitute a promising tool to analyze many-body effects in complex systems, with the advantage of providing a description of high order phenomena which can be tuned and can even be at the level of single variables or pairs. The applications here described show the effectiveness of this approach for multivariate data in physiology. In the big-data setting, evaluation of gradients of O-information remains an heavy computational burden, indeed for n variables even first order gradients require the estimation of entropy terms or order up to n: further work will be devoted to develop approximate approaches for the evaluation of gradients so as to make it feasible also for a large number of variables n.

## Data and code availability

fMRI data were provided by the Human Connectome Project, WU-Minn Consortium (Principal Investigators: David Van Essen and Kamil Ugurbil; 1U54MH091657) funded by the 16 NIH Institutes and Centers that support the NIH Blueprint for Neuroscience Research; and by the McDonnell Center for Systems Neuroscience at Washington University. The code for the evaluation of gradients of O-information can be found in https://github.com/tomscag/GOI/.

## Data Availability

The original contributions presented in the study are included in the article/supplementary material, further inquiries can be directed to the corresponding author.
